# MicroRNA Regulation of the Autotaxin-Lysophosphatidic Acid Signaling Axis

**DOI:** 10.3390/cancers11091369

**Published:** 2019-09-14

**Authors:** Mandi M. Murph

**Affiliations:** Department of Pharmaceutical and Biomedical Sciences, College of Pharmacy, University of Georgia, Athens, GA 30602, USA; mmurph@uga.edu; Tel.: +1-706-583-0216

**Keywords:** autotaxin, lysophosphatidic acid, miR-30c-2-3p, miR-122, miR-489-3p, miR-101-3p, miR-200c, miR-15b, miR-29a/b/c, and miR-21

## Abstract

The revelation that microRNAs (miRNAs) exist within the human genome uncovered an underappreciated mechanism of gene expression. For cells to regulate expression of their genes, miRNA molecules and argonaute proteins bind to mRNAs and interfere with efficient translation of the RNA transcript. Although miRNAs have important roles in normal tissues, miRNAs may adopt aberrant functions in malignant cells depending on their classification as either a tumor suppressor or oncogenic miRNA. Within this review, the current status of miRNA regulation is described in the context of signaling through the lysophosphatidic acid receptors, including the lysophosphatidic acid-producing enzyme, autotaxin. Thus far, research has revealed miRNAs that increase in response to lysophosphatidic acid stimulation, such as miR-21, miR-30c-2-3p, and miR-122. Other miRNAs inhibit the translation of lysophosphatidic acid receptors, such as miR-15b, miR-23a, and miR200c, or proteins that are downstream of lysophosphatidic acid signaling, such as miR-146 and miR-21. With thousands of miRNAs still uncharacterized, it is anticipated that the complex regulation of lysophosphatidic acid signaling by miRNAs will continue to be elucidated. RNA-based therapeutics have entered the clinic with enormous potential in precision medicine. This exciting field is rapidly emerging and it will be fascinating to witness its expansion in scope.

## 1. Introduction to MiRNAs

MicroRNAs (miRNAs) are endogenous, non-translated RNAs comprised of ~22 nucleotides that regulate gene expression [1,2]. Functionally, miRNAs bind to the 3′-untranslated region of many mRNAs and thereby silence gene expression by interfering with translation [3]. Through the creation of an mRNA-miRNA-protein complex, efficient translation is blocked. Therefore, a single miRNA can affect hundreds of cellular transcripts, suggesting enormous impact from the presence of these small nucleotides. Thus, it is not surprising that growing evidence indicates that dysfunction of miRNA expression influences tumorigenesis [2,4].

Concealed within the plant and mammalian genomes until their discovery in 1993 [5], miRNAs play a critical role in “fine-tuning” gene expression [6]. In contrast, miRNAs can also have aberrant roles when they are deregulated in cancer cells, thereby permitting unusual adjustments in gene expression. As a result, alterations in miRNAs modify the normal silencing of thousands of oncogene transcripts in a cell with potentially catastrophic consequences.

**Figure 1:**
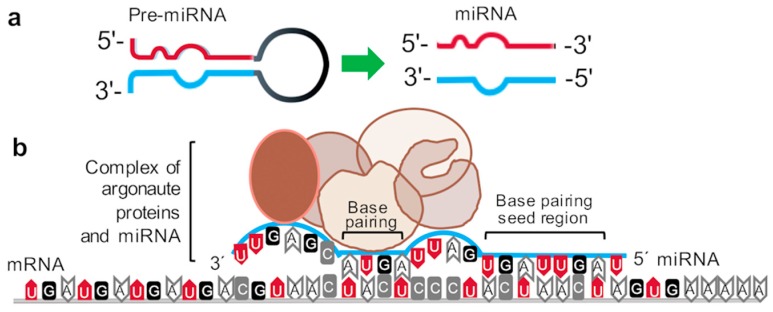
Schematic of the complex with mRNA–microRNA (miRNA)–argonaute proteins. (**a**) Mature miRNA strand processing simplified from the pre-miRNA hairpin loop structure. (**b**) The visual representation demonstrates the bulky complex formed by miRNA binding to mRNA, which reduces efficient transcript processing for protein translation. The miRNA 5′ end sequence “seed region” includes base positions 2–8, and is fundamental for miRNA–mRNA base pairing. In addition, base pairing is also likely among bases 13–16, as depicted in the schematic. These interactions destabilize the mRNA and inhibit transcript processing. Note that imperfect base pairing is expected among the other sequences in the miRNA strand and does not detract from its function. Argonaute proteins (brown-colored, oval shaped) are bound to the miRNAs during processing, which contributes to the bulky complex formation affecting translation.

During the processing and formation of miRNAs, the hairpin loop structure of miRNA is cleaved by Dicer into two strands, the “5p” and “3p”, formerly the major/guide and minor/passenger/* strand, respectively. As the names suggest, the 5p strand exists in a forward, 5′-3′ conformation, whereas the 3p strand exists in reverse, 3′-5′ conformation (Figure 1a). Both strands can directly target mRNAs and interfere with efficient transcription, although the 5p strand generally has a position of prominence because it is more abundant in the cell due to a loading bias into RNA-induced silencing complex (RISC) [7]. The fundamental binding interaction that regulates the transcriptional interference is largely dependent upon the base pairing between the seed region of the miRNA with the 3′-untranslated region of the mRNA (Figure 1b).

Current nomenclature describes miRNA strands as miR-# or less often, miR-#-5p, for the 5p strand and 3p for the other. Thus, a miRNA without denoting “5p” or “3p” after its number indicates that it is the major or 5p strand. For example, “miR-122” means this is the 5p strand, although it is seldom designated as miR-122-5p. The 3p strand must be written as “miR-122-3p”. The exceptions to this rule are lin-4 and let-7 (and its family members), the originally discovered miRNAs in *Caenorhabditis elegans* (*C. elegans*), prior to the establishment of conventional nomenclature [8,9].

## 2. Overview of Lysophosphatidic Acid Signaling and Autotaxin in Cancer

### 2.1. Lysophosphatidic Acid Signaling in Cancer

Lysophosphatidic acid is a phospholipid with a single carbon chain and phosphate group, which yields significant cellular outcomes in cancer, including viability, growth, motility, proliferation, invasion, and survival [10]. Normally, lysophosphatidic acid is found in circulation where it is significantly increased by activated platelets in response to wound healing and to aid the immune response [11,12]. While a poor biomarker on its own, it has historically been linked to cancer through its presence as an activating factor in the ascites fluid of ovarian cancer [13]. Indeed, lysophosphatidic acid was shown to diminish the ability of cisplatin to kill ovarian cancer cells, supporting a powerful ability to enhance cell viability and interfere with treatment [14]. Further studies corroborated the presence of lysophosphatidic acid in malignant effusions and other gynecologic cancers [11,15]. Concerning outcomes in cancer, among serous epithelial ovarian cancer patients, those with a transcriptional signature indicative of prevailing lysophosphatidic acid signaling in their tumor microenvironments have a worsened prognosis when compared to others [16].

Under malignant conditions, lysophosphatidic acid acts as an aberrant lipid mediator, which exacerbates signaling due to its role as an agonist and ligand for a family of G protein-coupled receptors. There are many reported receptors (some putative) for lysophosphatidic acid: LPAR1/Edg2 [17], LPAR2/Edg4 [16], LPAR3/Edg7 [18], LPAR4/GPR23 [19], LPAR5/GPR92/93 [20,21], LPAR6/P2Y5 [22], GPR87/95 (putative LPAR7) [23], PPARγ [24], LyPS2/P2Y10 (lysophosphatidyl serine and putative dual lysophosphatidic acid (LPA) and S1P receptor) [25], and receptor for advanced glycation endproducts (RAGE) [26]. Furthermore, there are other ligands, such as gintonin, that are agonists for the lysophosphatidic acid receptors [27]. This suggest that designing therapeutic targets for specific agonists or receptors will likely be futile if the goal is to inhibit signal transduction. Lastly, increasing receptor expression and/or lysophosphatidic acid production contributes to tumor progression in both ovarian [16,28,29,30] and breast cancer models [31,32]. Once again, suggesting that therapeutic targeting of this system is quite complicated and newer approaches are necessary for such an endeavor.

### 2.2. Autotaxin in Cancers

The identification of autotaxin in the conditioned medium of melanoma cells the supported involvement of the lysophosphatidic acid signaling network in cancer [33]. Autotaxin is a secreted enzyme in tissues and circulation that metabolizes lysophosphatidylcholine to yield lysophosphatidic acid [34,35,36]. It is a phospholipase and phosphodiesterase involved in both inflammation and cancer progression [31,33,34]. Alterations that enhance autotaxin expression correlate with renal cell carcinoma, breast cancer, ovarian cancer, bladder carcinoma, thyroid carcinoma, and glioblastoma multiforme [37,38,39]. Transgenic mice engineered to over-produce autotaxin have tumor formation after 12 months, mimicking human models of cancer development [32,40]. Additional studies suggest that serum measurements of autotaxin concentration may be useful as a biomarker for follicular lymphoma [41]. Within the tumor microenvironment, autotaxin is found where tumor cells are undergoing angiogenesis [42], due to its normal role in vasculogenesis [43,44]. In general, inhibiting autotaxin through chemical means or siRNA delivery will kill cells and prevent metastatic progression, in vitro and in vivo [34,42,45,46,47,48].

Autotaxin is the major producer of lysophosphatidic acid through the hydrolysis of the choline headgroup from lysophosphatidyl choline [35]. Although other enzymes complete reactions that may also yield lysophosphatidic acid, in the wound healing microenvironment of blister fluid, autotaxin activity was detected but not phospholipase A2 activity [49]. Thus, due to its critical role in regulating the levels of lysophosphatidic acid in circulation and wound healing, it is not surprising that this important enzyme has regulatory miRNA affecting its expression.

## 3. MiRNA as a Puppet Master of Lysophosphatidic Acid Signaling Events

Among tumor cells in malignant tissue, there is a global decrease of miRNA expression inside the tumor cells when compared to normal cells in adjacent tissue, implying roles for some miRNAs as tumor suppressors [50]. We and others have observed the export of miRNAs from cancer cells during tumorigenesis, which frequently facilitates signaling events beneficial to malignancy [40,51]. This section contains examples where this occurs, but signaling outcomes are dependent upon the functional consequences of individual miRNAs. For example, exportation of tumor suppressor miRNAs might occur, while oncogenic miRNAs, sometimes referred to as “oncomiRs”, might be upregulated and retained inside the cell.

### 3.1. Lysophosphatidic Acid Signaling Regulation

#### 3.1.1. MiR-30c-2-3p

The first published report between lysophosphatidic acid and miRNA described the role of miR-30c-2*, which is now denoted miR-30c-2-3p, and its actions as part of a feedback loop regulating signaling. Lysophosphatidic acid stimulation of ovarian cancer cells produces the transcription of numerous miRNAs, particularly a significant abundance of miR-30c-2-3p, which functionally reduces cell viability and proliferation and facilitates cell death [52]. The initial results characterizing the functional role of miR-30c-2-3p were confirmed by multiple groups, which emphasized an important role in cell cycle regulation [53,54,55,56]. MiR-30c-2-3p is included among miRNA signatures associated with apoptosis when upregulated [57].

Interestingly, miR-30c-2-3p is also expressed in response to other growth factors besides lysophosphatidic acid, including epidermal growth factor and platelet-derived growth factor, suggesting a broad regulatory function for regulating receptor-mediated signaling activation [52]. Further investigation into the mechanism of this miRNA revealed that miR-30c-2-3p inhibits the transcription of the cell cycle protein cyclin E1 [55,56], impacts NF-kB signaling through TRADD targeting [55], and directly targets RAB31 [58], BCL9 [52], and activating transcription factor 3 (ATF-3) [59]. Indeed, the expression of the transcription factor, ATF-3, is also immediately stimulated in response to lysophosphatidic acid. Thus, miR-30c-2-3p is part of a feedback loop that controls the transcriptional outcomes mediated by lysophosphatidic acid. [52,59] From a biological perspective, it is fascinating that the cell has adapted so many diverse and complex mechanisms to down-regulate receptor-mediating signaling activity stemming from the plasma membrane.

**Figure 2:**
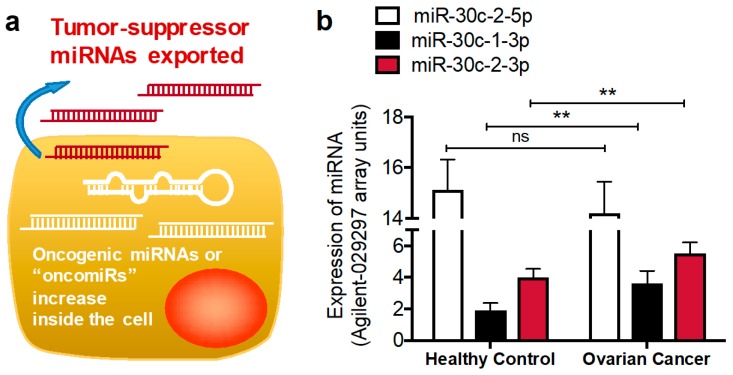
Potential mechanism of miRNA shifts in cancer cells and serum. (**a**) An overall increase in the abundance of miRNAs may occur in malignancy, but the localization is key to their function. (**b**) GEO Datasets was mined for miRNA expression in ovarian cancer patients. GSE48485 shows healthy serum (*n* = 5) versus serum from ovarian cancer patients (*n* = 5) with changes in miRNA-30c-2-3p and miR-30c-1-3p. ** *p* < 0.01, ns: miR-30c-2-5p. The graph also demonstrates the relative abundance difference between the major and minor miRNA strands of miR-30c-2.

These studies suggest that miR-30c-2-3p is a functional tumor suppressor that counteracts growth factor activation of the cell cycle and proliferation after signaling is initiated by the receptors. Thus, it downregulates signaling response outcomes. If true, then the presence of miR-30c-2-3p would be unexpected inside a tumor cell at the same quantity as a normal cell, particularly due to its role in proliferation and the cell cycle. Indeed, the cell has export mechanisms that facilitate the transfer of miRNAs out of the cell so that the intracellular amount of miR-30c-2-3p is different than the extracellular milieu (Figure 2a). In this example, oncogenic miRNAs that target critical regulatory proteins can be increased and retained, whereas tumor suppressor miRNAs are increasingly exported. Such an occurrence is observed upon examination of ovarian cancer patient serum (Figure 2b) [60].

#### 3.1.2. MiR-21

The first functional study of miR-21 described in neoplasia observed its strong over-expression among glioblastoma multiforme cells and tissues, which suggested a role as an oncogenic miRNA. The result of miR-21 suppression in glioblastoma cells causes caspase activation and associated apoptotic cell death. [61] Although this initial study did not indicate the direct target of miR-21, later work uncovered that among other transcripts, PTEN is a direct target and miR-21 thus modulates cancer cell growth and invasion through this interaction [62]. Since the PI3K/Akt pathway is often involved in the progression of ovarian cancer, it is consistent that miR-21 is increased in ovarian cancer tissues versus normal tissues [63].

**Figure 3:**
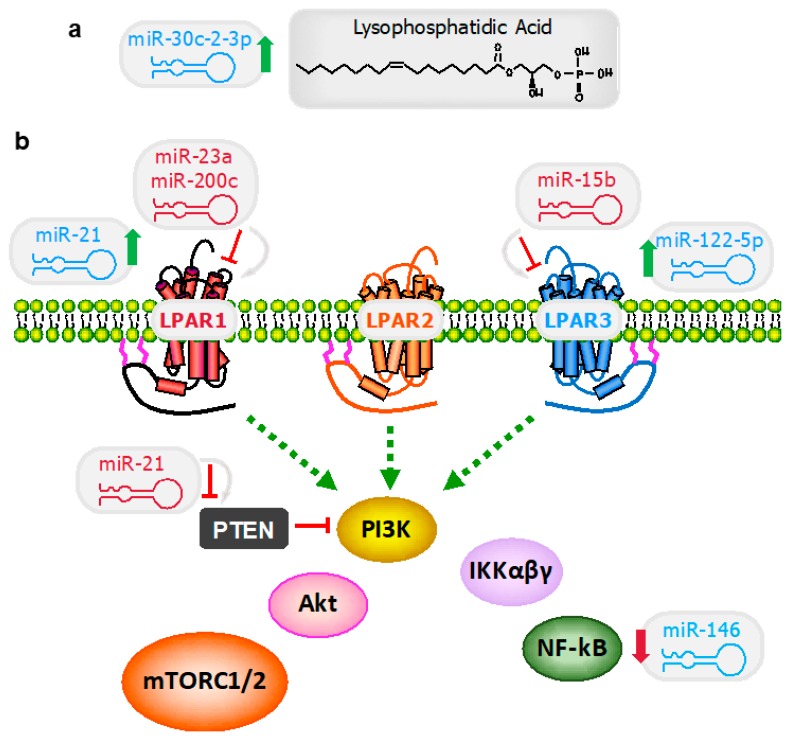
MiRNA regulation of the lysophosphatidic acid–autotaxin signaling axis. (**a**) Lysophosphatidic acid and other growth factors increase the expression of miR-30c-2-3p, which inhibits transcriptional events mediated as a consequence of receptor signaling. (**b**) Various miRNAs directly inhibit LPAR1, LPAR3, or PTEN (shown in red with gray arrows and red inhibitor lines pointing at their binding transcript partner). Alternatively, signaling through other receptors or pathways results in an increase or decrease of other miRNAs (shown in blue with arrows indicated an increase, green, or a decrease, red). See text for more details on these interactions.

Stimulation of MDA-MB-231 breast cancer cells with 1 μM of lysophosphatidic acid upregulated numerous miRNAs, including and especially miR-21 [64]. The authors recapitulated this response in MDA-BO2 and Hs578T breast cancer cells using 10 μM of lysophosphatidic acid, and siLPAR1 inhibited this effect. Thus, they concluded that the addition of lysophosphatidic acid sustains a 24-hour increase in miR-21 by 3- to 4-fold via signaling through the LPAR1. This is illustrated in the schematic showing miR-21 in blue next to LPAR1, which represents an indirect increase in the miRNA related to the signaling activation. In contrast, the red miR-21 next to PTEN indicates that this is a direct target of the miRNA which would impact all downstream signaling proteins as well (Figure 3).

#### 3.1.3. MiR-146a

MiR-146a is a quintessential example of a miRNA that directly targets transcripts of upstream proteins in signaling pathways, therefore indirectly blunting downstream signaling effects as a consequence as outlined herein. Foremost, lysophosphatidic acid activates signaling through the NF-kB pathway [65]. Additionally, miR-146a expression is altered in some gastric cancers and directly targets CARD10, COPS8, IRAK1, and TRAF6 [66,67]. As a result, miR-146a expression negatively impacts the receptor-mediated activation of NF-kB by lysophosphatidic acid. Therefore, without acting directly on NF-kB, miR-146a regulates its activity, by inhibiting the secretion of tumor-promoting chemokines and cytokines controlled by NF-kB [66,68].

### 3.2. Lysophosphatidic Acid Receptors

#### 3.2.1. MiR-122

The miRNA miR-122, or miR-122-5p, is most commonly recognized for its role in upregulating the replication of the hepatitis C virus RNA genome and RNA viral stability [69]. Under this scenario, as miR-122 binds to viral RNA, there is less miR-122 available overall, which negatively impacts normal liver function and increases the risk for hepatocellular carcinoma [70]. In a normal context, miR-122 was initially thought to be a liver-specific miRNA; however, hepatocytes are not necessarily the only cell type whereby it has a role, and many other cancer subtypes show its presence [51,70,71].

Signaling through the LPAR3 caused a surge in miR-122 production within melanoma cells and also exported as cargo into exosomes [51]. Delving into this mechanism, Byrnes et al. uncovered specifically that the Src homology 3 ligand-binding motif within the third intracellular loop of the receptor was responsible for the upregulation of miR-122. Although miRNAs have many targets, in this example, miR-122 targeted Wnt1 transcription, among others. In summary, miR-122 is another quintessential example demonstrating how miRNAs regulate lysophosphatidic acid stimulation of G protein-coupled receptor signaling.

#### 3.2.2. MiR-15b

MiR-15b is a member of the miR-15/16 family, which is well known to impact cell cycle progression and proliferation. The miR-15/16 family is involved in a feed-forward loop whereby the transcription factor, E2F, increases the expression levels of these miRNAs, which then directly inhibits cyclin E and modulates the cell cycle [72]. MiR-15b directly targets the BCL2 and WNT7A transcripts to modulate apoptosis and tumor progression [73,74]. Exogenous addition of the miR-15b mimic in ovarian cancer cells significantly reduces the mRNA and protein of LPAR3 by ~70%. In addition, the mRNA and expression of downstream signaling proteins, PI3K and Akt, is also impacted. [75] This again demonstrates how miRNAs can modulate receptor-mediated signaling.

#### 3.2.3. MiR-200c

The family of miR-200 represents a very important group of miRNAs that regulate the epithelial-to-mesenchymal-transitioning or the EMT process in cancer [76]. For example, miR-200c helps to maintain the epithelial phenotype through targeting ZEB1 in a feed-forward loop triggered by ZEB1 suppression of miR-200c transcription [77,78]. Consistent with this work is the observation that miR-200c’s loss indicates a shift to the mesenchymal phenotype [79]. Thus, it is well established that through this molecular shift, the miR-200 family affects invasion, morphological plasticity, and ultimately, the metastatic process [80]. Interestingly, other work surrounding contractile physiology highlighted additional direct targets of miR-200c, including RhoA kinase and the LPAR1 [81].

RhoA and LPAR1 are an interesting pair for miR-200c targeting because the LPAR1 couples to the Gα_12/13_-linked RhoA kinase pathway to mediate transient contraction of the cytoskeleton. This effect peaks after 2–3 minutes of stimulation with 1 μM of lysophosphatidic acid to activate cell motility [82]. Furthermore, mutation of the LPAR1 phosphorylation site at Thr-236 which mediates signal transduction and attenuation exhibited an elevation in the basal activation of RhoA [29]. This data further demonstrates the interaction between the LPAR1 and RhoA at the residue that is critical for dampening receptor signaling. Thus, miR-200c is targeting a specific process in cells by mediating the abundance of the receptor and the subsequent signaling GTPase protein, adding a layer of complexity and regulation to the signal transduction.

#### 3.2.4. miR-23a

Although the observation occurs outside of a cancer system, there is at least one example of a receptor reciprocal regulation using miRNA as an intermediate. Lysophosphatidic acid (5 μM) or the selective agonist 1-oleoyl-2-methyl-sn-glycero-3-phosphothionate (OMPT) (1 μM) addition to cardiomyocytes for 1–48 hours significantly increased the expression level of miR-23a. Exogenous addition of pre-miR-23a to cells reduced the relative mRNA level of LPAR1 approximately 50%. However, when the authors used siRNA to inhibit LPAR1, there was no significant impact on miR-23a induction by lysophosphatidic acid. It was only when siRNA for LPAR3 was added that the increased expression of miR-23a was blocked [83]. Taken together, these data suggest that signaling activation of the LPAR3 reduces the expression of the LPAR1 by half due to an increase of miR-23a expression, at least in cardiomyoctes. It is unclear how the control exerted by LPAR3 may impact other cellular systems. If this is widely applicable, then it may suggest LPAR3 is the ultimate puppet master through miRNA modulation.

### 3.3. Autotaxin

#### 3.3.1. MiR-29a/b/c

**Figure 4:**
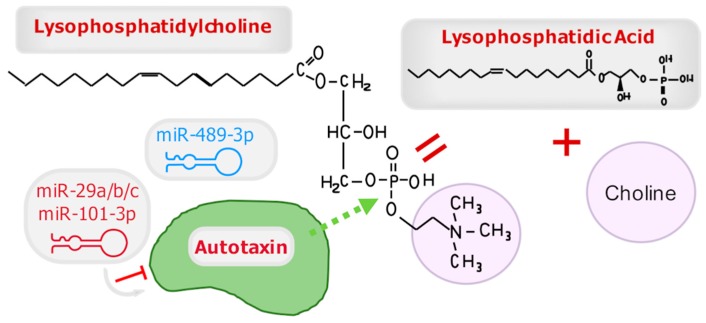
The miRNA regulation of autotaxin. The schematic shows where lysophosphatidylcholine is hydrolyzed by autotaxin to yield lysophosphatidic acid and choline. Several miRNAs are strongly predicted (miR-29 family) or bona fide (miR-101-3p) binding partners of autotaxin mRNA (miRNA shown in red with gray arrows pointing at their transcript target), resulting in its transcriptional silencing. Additionally, autotaxin expression indirectly facilitates the exportation of miR-489-3p out of cells/tissues and into serum, likely as part of a larger feedback mechanism observed in malignancy. MiRNAs indirectly affected by autotaxin are indicated in blue.

The first publication to address autotaxin and miRNA regulation in a clinical context described the miR-29 family members miR-29a-3p, miR-29b-3p, and miR-29c-3p, in chronic obstructive pulmonary disease or COPD. These miRNA candidates were predicted by TargetScan 7.1 version (www.targetscan.org) as miRNAs that contained 6–8 base pair sites that matched with the seed region of autotaxin. This work demonstrated that the autotaxin signaling axis was attenuated in males, but not females; furthermore, this attenuation corresponded to a significant increase in miR-29a-3p and miR-29b-3p in the bronchoalveolar lavage cells from COPD patients, and miR-29b-3p and miR-29c-3p in the bronchial epithelial cell brushings [84]. In conclusion, although the work did not yield a direct binding effect, it does suggest that autotaxin is likely associated with the mechanism and/or prevalence of COPD in one sex as part of an altered metabolic pathway (Figure 4). This is quite interesting and consistent with the observation that females and males have significant differences in the total concentration of lysophosphatidic acid in plasma and serum [85].

#### 3.3.2. MiR-489-3p

A study analyzing miRNAs found in circulating exosomes of mice with enhanced autotaxin expression observed numerous changes in the miRNome, again suggesting that miRNAs are puppet masters of lysophosphatidic acid signaling events. In addition to the multitude of altered miRNAs found in circulation between wild-type FVB/N mice and autotaxin-overexpressing FVB/N mice, some miRNAs identified mice at high risk for cancer. The high-risk animals that went on to develop cancer also had aberrant miRNA regulation consistent with an increase among specific signaling pathways in cancer, including FoxO, MAPK, PI3K-Akt, Ras, and TGF-β signaling. A miRNA biomarker in serum for this cohort was miR-489-3p, which was exported out of the tumor cells. This miRNA was shown to inhibit MEK1 through a 7-mer, base-pairing sequence. Correspondingly, tumor tissues demonstrated abundant expression of MEK1 protein and autotaxin. Therefore, while miR-489-3p does not have a direct impact on autotaxin translation and instead targets MEK1, increased autotaxin overrides this effect, suggesting another potential feedback loop that modulated in cancer [40].

#### 3.3.3. MiR-101-3p

Various cancer subtypes, such as breast, colon, and glioblastoma cells, demonstrate an inverse correlation between the expression of autotaxin and miR-101-3p. Further exploration by Wang et al. into the putative binding site between these RNA molecules demonstrated a 7-mer binding site in the miRNA seed region that would base pair with the mRNA of autotaxin. When the site was mutated on autotaxin, miR-101-3p lost the ability to reduce the luciferase reporter activity, which confirmed the interaction. A particularly interesting element in this report was the authors’ demonstration that miR-101-3p out-performed the ability of targeted siRNA against autotaxin to inhibit cell growth/confluence. Since only exogenous miR-101-3p addition was able to inhibit this effect <50%, it shows a more effective outcome using miRNA over siRNA, at least in this example [86].

## 4. MiRNAs as Therapeutics

Numerous RNA-based therapeutics are under investigation in preclinical models as well as phase 1 and 2 clinical trials [87,88], and others have recently received regulatory approval. There is a wide range of possibilities for therapeutic design in this area, including miRNA “mimics”, antagomiRs or anti-miRs, Piwi-interacting RNAs (piRNAs), and long, non-coding RNAs (lncRNAs). The application will obviously depend on the cause of the disease and whether the RNA molecule’s target is aberrantly-acting to drive pathology or absent as a result of a suppressive function from the pathology. As a hypothetical example, a targeted down-regulation of autotaxin and lysophosphatidic acid receptors that mediate pain might be an alternative approach to modulate this response.

In August 2018, the U.S. Food and Drug Administration (FDA) approved the first RNA-based therapeutic, patisiran (Onpattro^®^, Cambridge, MA, USA) by Alnylam Pharmaceuticals, for treating a hereditary peripheral nerve disease (fda.gov). Shortly thereafter, a similar RNA-based interference drug, inotersen (Tegsedi^®^, Boston, MA, USA) by Akcea Therapeutiscs and Ionis Pharmaceuticals was also approved by the FDA. The goal of these agents is to reduce the buildup of the transthyretin protein, for which accumulation of abnormal transthyretin deposits leads to muscle atrophy, reduced pain sensations, and reduced mobility in the body, which are ultimately responsible for causing disease. Parisiran is an intravenously-administered agent, whereas inotersen is a weekly subcutaneous injection.

Although the previous therapeutic examples are outside the field of oncology, the dearth of approvals for malignant conditions is expected to change. Companies like Regulus Therapeutics, miRagen Therapeutics, and others have numerous agents in their pipelines to enter the oncology market. As examples, their pipelines include therapeutics to target glioblastoma multiforme by inhibiting oncogenic miR-10b and target various types of lymphomas and leukemias with cobomarsen (an inhibitor of miR-155) [89,90,91]. In summary, the discovery and unique abilities of miRNAs has made this an exciting time for designing treatments for rare diseases and tailoring other miRNAs or anti-miRs for precision therapeutics.

## 5. Conclusions

This review highlights major findings on the regulation of lysophosphatidic signaling-mediated events and the impact on gene expression by miRNAs. These highlights are summarized in Table 1. It also illuminates several examples of miRNA-associated feed-back and feed-forward loops that regulate signaling. To reiterate an example of this, lysophosphatidic acid-mediated receptor activation initiates a cascade of signaling events, including an increase in activating transcription factor 3 (ATF-3), which upregulates miR-30c-2-3p. In response, miR-30c-2-3p then inhibits the expression of other transcripts induced in response to lysophosphatidic acid, including ATF-3. Understanding these loops and miRNA regulation has the potential to aid strategic design of precision therapeutics that could impact rare diseases and malignancy. This is an incredibly exciting time to witness the marriage of genetic research with therapeutics that is anticipated to expand exponentially.

## Figures and Tables

**Table 1:** Summarizing miRNA content included in the text.

miRNA	Target	Alteration
miR-15b	BCL-2, LPAR3, WNT7A	LPAR3 mRNA and protein
miR-21	PTEN	Upregulated by LPAR1 signaling through the PI3K pathway
miR-23a	LPAR1	Reduced LPAR1 through LPAR3 in cardiomyocyte hypertrophy
miR-29a/b/c	ATX/ENPP2	ATX mRNA and protein
miR-30c-2-3p	ATF3, BCL-9, CCNE1, HIF2A, RAB31, TRADD, XBP1	Lysophosphatidic acid signaling, cell cycle and death
miR-101-3p	ATX/ENPP2	ATX mRNA and protein
miR-122-5p	WNT1	Lysophosphatidic acid signaling through LPAR3
miR-146a	COPS8, CAR10	Modest reduction of NF-kB activation and cytokine mRNA by 25 μM lysophosphatidic acid
miR-200c	LPAR1	Cell contraction
miR-489-3p	MEK1	Feedback loop with autotaxin signaling
